# Novel Immune Check-Point Regulators in Tolerance Maintenance

**DOI:** 10.3389/fimmu.2015.00421

**Published:** 2015-08-18

**Authors:** Yanxia Guo, Adele Y. Wang

**Affiliations:** ^1^Merck Research Laboratories, Palo Alto, CA, USA

**Keywords:** B7, butyrophilin, immune tolerance

## Abstract

The great success of anti-cytotoxic lymphocyte antigen 4 (CTLA4) and anti-programed cell death protein 1 (PD1) in cancer treatment has encouraged more effort in harnessing the immune response through immunomodulatory molecules in various diseases. The immunoglobulin (Ig) super family comprises the majority of immunomodulatory molecules. Discovery of novel Ig super family members has brought novel insights into the function of different immune cells in tolerance maintenance. In this review, we discuss the function of newly identified B7 family molecules, B7-H4 and V-domain Ig Suppressor of T cell Activation (VISTA), and the butyrophilin/butyrophilin-like family members. We discuss the current stages of immunomodulatory molecules in clinical trials of organ transplantation. The potential of engaging the novel Ig superfamily members in tolerance maintenance is also discussed. We conclude with the challenges remaining to manipulate these molecules in the immune response.

## Introduction

The immune system consists of various cell types to mount both innate and adaptive immunity against pathogens. As an important component of adaptive immunity, T cell activation requires three signals. Signal 1 is acquired through the interaction between T cell receptor (TCR) and peptide-loaded major histocompatibility complex (MHC) molecules. Signal 2 is induced through the interaction between co-stimulatory/co-inhibitory molecules on antigen-presenting cells (APC) and their receptors on T cells, thus enhancing/inhibiting T cell activation. Signal 3 is mediated by cytokines, such as IL2 to promote T cell proliferation. Co-stimulatory/co-inhibitory molecules are the most well-studied molecules and fall into immunoglobulin (Ig) super family members, including B7 family and tumor necrosis factor (TNF) family proteins. These molecules play essential roles in maintaining the balance between sufficient immune response against pathogenic antigens (Ag) as well as limited response against self-antigens or allogenic transplant graft. We identify this family of molecules as “immune modulatory molecules.” The potential receptors and ligands for many novel molecules remain unknown and may serve as both receptors and ligands in different cells. In this review, we define the potential receptors/ligands of these novel molecules as “binding partners” for simplicity.

Over the past decade, a large number of B7 and TNF family molecules have been discovered and actively evaluated in clinic. The clinical success of anti-CTLA4 (1, 2) and anti-PD1 blocking antibody (Ab) (3, 4) in various cancers encourages more active research and drug development effort. This effort aims to enhance co-stimulatory or dampening co-inhibitory molecules effect to achieve robust anti-tumor immune response. In parallel, studies are also carried out to dampen the immune response by enhancing the effect of co-inhibitory molecules in treatment of autoimmune or inflammatory diseases (5–7). In addition, organ transplantation is still a great challenge in the clinic as the allogenic transplant rejection is a common problem in patients. There is great need for inhibiting the host rejection of donor organs (8). Undoubtedly, the discovery of new immune modulatory molecules has offered new opportunities to manipulate the host immune system for tolerance maintenance and graft acceptance. In this article, we review recent discoveries of novel Ig B7 family members VISTA and B7-H4. We also review the butyrophilin (BTN) family members, namely “old molecules with novel immunomodulatory functions.” Learning from the on-going clinical trials on well-described molecules, we discuss the potential application of these new molecules.

## B7 Family

B7 family proteins are among the best-described immunomodulatory molecules so far. This family consists of both co-inhibitory and co-stimulatory molecules. This family includes B7.1 (CD80), B7.2 (CD86), CD28, CTLA4, PD1, PD1 ligand (PDL1), PDL2, B7RP1 (ICOSL), B7-H3, B7-H4, and VISTA. The growing list of B7 family members offers great opportunities to modulate immune response therapeutically in treating different diseases. This family of molecules was reviewed elsewhere thoroughly (9, 10). In the following section, we will discuss the progress in understanding two new molecules in the family, B7-H4 and VISTA.

## B7-H4

B7-H4 (B7x, B7S1, VTCN1), identified in 2003 (11), is a highly evolutionarily conserved molecule that shares 87% amino acid identity between human and mouse. Mature B7-H4 is a 50–80 kDa transmembrane protein with one IgV domain and one IgC domain. The mRNA expression of B7-H4 is found on both lymphoid and non-lymphoid tissues. However, B7-H4 protein expression is more restricted and not expressed on *ex vivo* human immune cells (T, B, DC, and monocytes), but can be induced after *in vitro* stimulation. Endocrine cells in pancreas have also been shown to express a moderate level of B7-H4. Signaling through B7-H4 pathway leads to the inhibition of TCR-mediated CD4^+^ and CD8^+^ T cell proliferation, cell-cycle progression, and IL-2 production (11–13). In experimental autoimmune encephalomyelitis (EAE) model, blocking B7-H4 on host cells by a monoclonal antibody accelerates T cell responses and enhances disease severity (11). However, B7-H4-deficient mice only mount mildly increased Th1 response and intact cytotoxic T-lymphocyte reactions against viral infections (14). The study indicated that B7-H4 is a negative regulator of T cell activation but only plays a minor role in fine-tuning T cell immunity. However, elevated soluble form of B7-H4 in the serum of rheumatoid arthritis (RA) patients is associated with increased disease severity. In a collagen-induced arthritis mouse model, both overexpression of soluble B7-H4 and genetic deletion of B7-H4 enhanced disease severity, due to enhanced B cell, T cell, and neutrophil response (15). This severity suggests that soluble B7-H4 acts as a decoy molecule to block the inhibitory function of B7-H4. Further confirmation of the correlation between soluble B7-H4 and disease severity in RA patients may indicate a new target pathway in autoimmune diseases. Although soluble B7-H4-immunoglobulin (B7-H4-Ig) fusion protein binds to activated T cells, the binding partner for B7-H4 has not been identified and does not bind to known CD28 family members, such as CD28, CTLA4, ICOS, and PD1.

**Figure 1:**
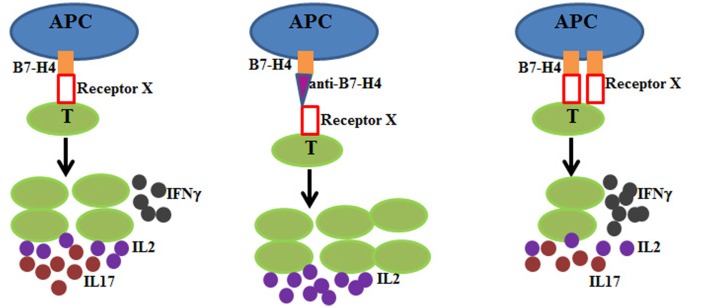
**B7-H4 is a co-inhibitory molecule on APC (blue) and interacts with its unknown receptor (receptor X) on T cells (green) to deliver negative signaling during T cell activation**. Left: in the presence of B7-H4-receptor X interaction, T cells proliferate at a lower rate and produce lower amount of IL2 (purple), lower IFNγ (black), and higher IL17 (red). Middle: antagonistic anti-B7-H4 (magenta triangle) blocks the interaction between B7-H4 and receptor X. T cells proliferate at a higher rate and produce more IL2. Right: B7-H4 overexpression leads to lower T cell proliferation, lower IL2 and IL17 production, and higher IFNγ production.

On the other hand, overexpression of B7-H4 on the pancreatic islets can protect mice from T cell-mediated autoimmunity and these islet allograft increased survival after transplantation (16). The immunosuppressive function of B7-H4 has also been investigated in experimental type I diabetes models using B7-H4-Ig. Pre-diabetic non-obese diabetes mice treated with B7-H4-Ig displayed lower incidence of diabetes by suppressing immune infiltrates into the islets (17). Lee et al. found that B7-H4-Ig treatment decreased Th17 cell whereas promoting CD4^+^IFNγ^+^ T cells development (18). These findings suggest a possible role of B7-H4 in maintaining the balance between Th1 and Th17 cells. However, more studies are warranted to verify this observation and elucidate the molecular mechanism. The impact of B7-H4 on T cells is summarized in Figure 1.

In addition to the direct impact of B7-H4 inhibition on T and B cell immune response, B7-H4 also acts as a negative regulator for neutrophil response. B7-H4-deficient mice are more resistant to infection by *Listeria monocytogenes* due to augmented neutrophil accumulation (19). Within 3 days after infection, bacterial colonies in liver and spleen are significantly lower in B7-H4-deficient mice than controls, concomitant with increased number of neutrophils in the spleen of infected mice. B7-H4 inhibits the growth of bone marrow-derived neutrophil progenitors *in vitro*, demonstrating an inhibitory function of B7-H4 in neutrophil expansion. Enhanced neutrophil growth in B7-H4-deficient mice also occurs in the background of recombination activation gene (RAG) knock out mice, independent of adaptive immunity. The inhibition of neutrophil expansion shows that B7-H4 directly regulates innate immunity. Furthermore, Pawar et al. discovered B7-H4 expression on kidney resident cells and could be induced further with inflammatory stimuli. In an antibody-mediated nephritis model, B7-H4-deficient mice showed more T, B, macrophage, and neutrophil infiltration into kidney. In addition, macrophages in B7-H4-deficient mice demonstrate a more inflammatory phenotype. Whether B7-H4 modulates other immune cells remains unknown.

## V Domain-Containing Ig Suppressor of T-Cell Activation

V domain-containing Ig suppressor of T-cell activation (VISTA), also named as PD1 homolog (PD1H), is expressed in both hematopoietic and non-hematopoietic tissues. Among immune cells, VISTA is highly expressed on mature CD11b^high^ myeloid-derived APCs and to a less extent on CD4^+^, CD8^+^, and Tregs (20). VISTA is a 55- to 65-kDa type I Ig membrane protein with the extracellular domain homologous to PD-L1. *In vitro*, VISTA-Ig inhibits T cell activation, proliferation, and production of cytokines, such as IL2 and IFNγ, during anti-CD3 activation. VISTA overexpression on APC also suppressed T cell proliferation, which was rescued by neutralizing anti-VISTA monoclonal Ab (13F3). More importantly, VISTA overexpression on tumor cells overcame the protective anti-tumor immunity. These studies collectively suggest that VISTA on APC delivers negative signaling to T cells during activation via a yet-to-identify binding partner on T cells. The same Ab administration *in vivo* exacerbated disease progression in a passive EAE model. Further studies demonstrated that mice deficient in VISTA display normal hematopoietic development. These mice do not succumb to obvious organ-specific autoimmune diseases, despite chronic inflammation in multiple organs over age. Consistently, spontaneously activated T cells accumulate and produce inflammatory cytokines and chemokines in aged VISTA-deficient mice (21). 2D2 T cell receptor mice overexpress T cell receptor that recognizes myelin oligodendrocyte glycoprotein (MOG_35–55_). VISTA deletion in 2D2 transgenic mice significantly enhanced EAE severity. Disease progression is due to an increase in activated encephalitogenic T cells in the periphery and greater infiltration into the central nervous system. The analysis of both VISTA-Ig and genetic ablation demonstrated that VISTA is a negative check-point regulator of T cell activation. Blocking VISTA’s function promotes the pro-inflammatory cytokines production and enhances autoimmune diseases development under susceptible conditions. However, another anti-VISTA Ab seemed to inhibit the progression of acute graft-versus-host-diseases (GVHD). This inhibition indicates the agonistic effect of this Ab in the regard of suppressing T cell activation (22).

**Figure 2:**
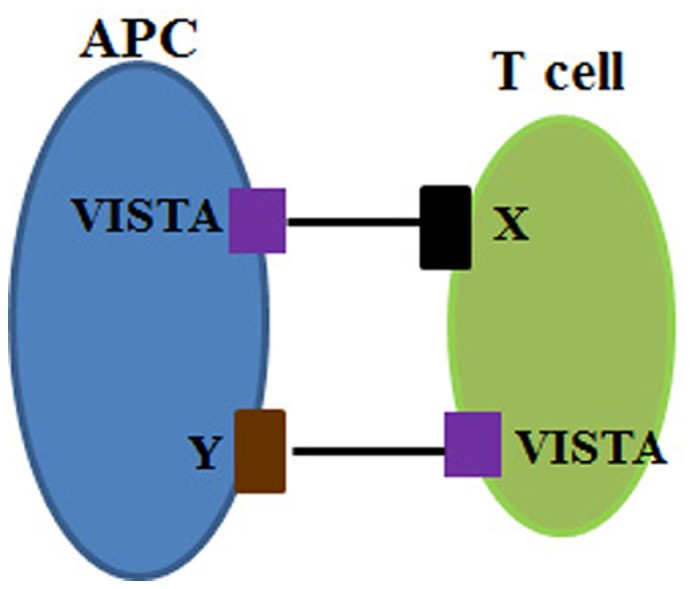
**VISTA is a co-inhibitory molecule on both APC (blue) and T cells (green)**. VISTA is expressed on APC and interacts with the partner (receptor X) on T cells. VISTA can also be a co-inhibitory receptor on T cells and binds to its unknown ligand (ligand Y) on APC. Both interactions deliver negative signaling to T cell activation.

Even though the binding partner of VISTA remains unknown, Flies et al. demonstrated that VISTA is a co-inhibitory receptor on CD4^+^ T cells. VISTA^−/−^ CD4^+^ T cells showed stronger Ag-specific proliferation and cytokine production than wild-type CD4^+^ T cells. In an APC-T co-culture study, VISTA deficiency on both APC and T cells resulted in the strongest proliferation response, whereas VISTA-sufficient APC and T cells generated the poorest response. VISTA deficiency on either APC or T cells yielded an intermediate response. Taken this data together with previous studies (20), VISTA negatively regulates T cell immunity via direct impact on T cells by engaging different receptor/ligand (illustrated in Figure 2). In a murine model of acute hepatitis, a different agonistic VISTA Ab administration suppressed CD4^+^ T cell and NK cell-mediated acute inflammation. The data suggested that VISTA^−/−^ mice showed survival benefits during GL261 glioma growth, mediated by enhanced CD4^+^ T cell anti-tumor immunity (22). The bidirectional inhibition of VISTA on APC and T cells suggests that VISTA is a valuable target for immunological diseases. The studies in mice are reinforced by characterization of human VISTA expression and function (23). More studies are needed to characterize the correlation between VISTA expression and autoimmune diseases, such as RA, to establish the rationale for targeting VISTA in clinic. Moreover, it is worthwhile evaluating the potential of agonistic anti-VISTA Ab in tolerance induction during organ transplantation given the preclinical evidences in GVHD. Furthermore, the identification of VISTA’s binding partner will certainly offer great insight into the molecular mechanism of VISTA signaling and ensure better understanding of agonistic Ab safety in development.

## Butyrophilin Family in Immune Tolerance

As B7 family member proteins, BTN family members have extracellular IgV and IgC domain, but most BTN family members have an intracellular B30.2 domain. Therefore, BTN family members belong to the Ig superfamily. Since the discovery of BTN family members in the regulation of immune response, butyrophilin-like (BTNL), family members have also been identified in both human and mouse genome. The extracellular structural similarity between BTN/BTNL and B7 family suggested that BTN/BTNL family molecules may have immune regulatory functions as B7 family members. In this section, we discuss the genomic distribution, function, and clinical evidence between BTN/BTNL family proteins and immune tolerance.

### Genomic distribution of BTNs

The first BTN member was discovered in milk in which BTN subfamily 1 member A1 (BTN1A1) is essential for the secretion, lactation, and stabilization of milk-fat globules (24–26). Further studies discovered more BTN proteins with immune-regulatory functions. Thus far, 13 human BTNs and BTNLs have been described. Human BTNs include BTN1A1, BTN2A1, BTN2A2, BTN2A3, BTN3A1, BTN3A2, and BTN3A3 (27). Human BTNLs include BTNL2, BTNL3, BTNL8, BTNL9, BTNL10, and SKINT-like (SKINTL). In mice, 11 genes have been identified to encode BTN and BTNL molecules. These include BTN1A1, BTN2A2, BTNL1, BTNL2, BTNL4, BTNL5, BTNL6, BTNL7, BTNL9, BTNL10, and SKINT. The majority of these proteins are diverse between human and mice, making it difficult to study their function. As mentioned above, the BTN family members have extracellular IgV and IgC domains as B7 family proteins (28–31). Extracellular IgV and IgC domains are essential for the interaction among B7 family members. However, it remains unknown whether BTN/BTNL family proteins bind to their counter-receptors via the IgV and IgC domain. In addition, it remains unknown how intracellular B30.2 domain binds to other intracellular signaling proteins to deliver the function of BTNs and BTNLs. Regarding the expression pattern of BTN/BTNL family molecules, there is some inconsistency among existing data when comparing mRNA with protein expression. Abeler-Dorner et al. summarized the mRNA and protein expression of each BTN/BTNL family member in another review (27).

### Function of butyrophilin in T cell immunity

The wide distributions of certain BTN/BTNLs determine the functions of these molecules in different immune cells. As BTN/BTNLs are expressed on APCs or T cells, studies have been carried out to determine the function of these molecules in the T cell immunity *in vitro* and *in vivo*. In this section, we will focus on the function of these “old molecules” in regulation of T cell immunity, in the regard of both conventional αβ T cells and γδ T cells.

#### Butyrophilin in Regulation of Conventional T Cell Response

As B7 family members, some BTN/BTNLs are co-inhibitory whereas others are co-stimulatory in the regard of conventional T cell function regulation. The function characterization of BTN/BTNL family members depends on the administration of recombinant protein and antagonistic/agonistic Ab in T cell activation, differentiation, and cytokine production *in vitro* and diseases progression *in vivo*. In the following section, we will focus on the delineation of co-inhibitory and co-stimulatory function of various BTN/BTNL family members.

##### BTNs as co-inhibitory molecules

Nguyen et al. first demonstrated the co-inhibitory role of mouse BTNL2 in T cell immunity (32). Mouse BTNL2-Ig fusion protein inhibited anti-CD3- and anti-CD28-stimulated CD4^+^ T cell proliferation and IL2 production via reducing activating protein-1 (AP-1), nuclear factor of activating T cells (NFAT), and NF-κB activity. Arnett et al. further illustrated the upregulation of BTNL2 in inflamed colons in the mouse Mdr1a-deficient model of inflammatory bowel diseases. BTNL2 conveys its inhibitory signal also by counteracting the co-stimulatory effect of B7RP1 (ICOS ligand) on T cell activation (33). No binding partner has been successfully identified as BTNL2 failed to bind known B7 family proteins, such as CD28, ICOS, PD1, CTLA4, and HVEM.

Further studies elucidated the similar co-inhibitory function of murine BTNL1 (34). *In vitro* studies showed that BTNL1-Ig fusion protein suppressed low-concentration anti-CD3-induced T cell activation and IL2 production by cell-cycle arrest. Like BTNL2, BTNL1 does not bind CD28, CTLA4, triggering receptor expressed on myeloid cells (TREM)-like transcript 2 (TLT-2, TREML2), B7-H3, B7-1, or other B7 family members. Antagonistic anti-BTNL1 Ab-induced strong Th2 response after keyhole limpet hemocyanin (KLH) and complete Freund’s adjuvant (CFA) immunization *in vivo*, demonstrated by enhanced T cell proliferation and higher production of Th2 cytokines (IL-4, IL-5, and IL-13), but lower IFNγ. Antagonistic anti-BTNL1 Ab treatment also exacerbated EAE via promoting Th17 response. Similarly, α-BTNL1 Ab exacerbated Th2-mediated allergy response. These studies indicate that BTNL1 may fine tune Th1 versus Th2 and Th17 development. No potential BTNL1 binding partner has been identified so far.

Human BTN3A1, also known as CD277, is highly upregulated in myeloid DCs and macrophages by vascular endothelial growth factor (VEGF) and CCL3 in human ovarian cancer. BTN3A1 inhibited T cell proliferation and Th1 cytokine production through preventing the upregulation of cellular caspase-8 (FLICE)-like inhibitory protein (cFLIP) and the consequently enhanced caspase-8 activity. Caspase-8 activity is necessary to initiate the activation of NF-κB and promote T cell proliferation (35). A recent study, however, extended the function of BTN3A1 beyond the co-inhibition to presenting phosphorylated antigen (discussed in more details below).

Even though the preclinical studies highlighted the importance of these co-inhibitory BTN/BTNLs in inflammatory diseases and possibly graft tolerance, key issues remain for therapeutic targeting of these molecules. First, little is known regarding how the negative signaling to T cell activation is conveyed by the engagement with their potential binding partners. The understanding in this regard will facilitate the identification of pharmacological kinetics (PK) and pharmacological dynamics (PD) markers during therapeutic Ab screening. Second, no potential binding partners for these molecules have been identified. To enhance the negative effect of the molecules during APC–T cell interaction, it is essential to minimize unwanted effects by activating potentially complex signaling pathway in T cells. More studies are needed to gain better understanding of the function of these co-inhibitory molecules in various T cell subsets differentiation and other immune cells. This will help to determine the specific need of the Ab isotype or the format of BTN/BTNL-Ig fusion protein to deliver the most effective drug with least toxicity.

##### BTNs as co-stimulatory molecules

Among BTN/BTNL family members, BTNL8 and BTN3A2 are co-stimulatory molecules. There is no ortholog of human BTNL8 in mice. BTNL8 mRNA is highly expressed on neutrophils rather than T cells or myeloid cells. BTNL8 seems to bind resting instead of activated T cells. The administration of BTNL8-Ig enhanced anti-CD3-stimulated human CD4^+^ T cell proliferation and cytokines production, such as IFNγ, TNFα, IL8, and IL10. This study indicated that BTNL8-Ig functions at very early stage of T cell activation (36). Surprisingly, BTNL8-Ig fusion protein enhanced Ovalbumin and alum-stimulated primary Ab response in mice. However, no studies have illustrated the reason for cross-species activity of BTNL8-Ig. Therefore, the binding partner of human BTNL8 may share high homology with its ortholog in mice. BTNL3 shares high sequence similarity BTNL8 but is expressed mainly in neutrophils. No known function of BTNL3 has been reported. In a population genetics study, Aginel et al. identified a *BTNL8–BTNL3* deletion leading to a new BTNL8*3 fusion protein (37). The sequence between intron 4 of BTNL8 and intron 4 of BTNL3 is deleted, leading to no coding sequence changes in BTNL8–BTNL3. Thus, the fusion protein may still be intact. Gene-expression analysis in lymphoblastoid cell lines showed that *BTNL8–BTNL3* deletion is correlated with TNF and ERK1/AKT pathways, the key players in immune regulation. However, function analysis needs to be done. Further studies are warranted to elucidate the correlation between *BTNL8–BTNL3* deletion and inflammatory diseases in patients.

BTN3A2 was recognized as a co-stimulatory molecule from correlation studies in cancer patients. Earlier studies suggested that high BTN3A2 mRNA expression was associated with a good prognosis in relation to disease-free and overall survival in a cohort of 55 epithelial ovarian cancer (EOC) patients (38). Further studies in a larger cohort of 199 high-grade EOC patients confirmed that the protein expression of BTN3A2 in ovarian cancer tissues is positively correlated with the intraepithelial infiltration of CD4^+^ and CD8^+^ T cells (39). The validation of BTN3A2 as a prognostic marker for EOC indicated that BTN3A2 may modulate the infiltration of immune cells and thus the anti-cancer immunity. To better understand whether BTN3A2 can be a therapeutic target in cancer, inflammatory diseases, or transplant tolerance, the function of BTN3A2 in specific immune cells warrants further studies.

#### BTN as Antigen Presentation Molecule

A very recent study demonstrated the function of BTN3A1 beyond the co-inhibitory and co-stimulatory roles in immune modulation (40, 41). Human Vγ9Vδ2 T cells are activated by phosphorylated antigens. Theses antigens include host isopentenylpyrophosphate (IPP) and microbial (E)-4-hydroxy-3-methyl-but-2-enyl pyrophosphate (HMBPP). Vavassori et al. discovered that recombinant BTN3A1 specifically stimulated human Vγ9Vδ2 T cells in the presence of IPP. This effect is independent of CD1d molecule. BTN3A1 binds to IPP at a stoichiometry ratio of 1:1 at a lower affinity (*K*_d_ = 10^−6^ M) than the binding of MHC to peptides (*K*_d_ = 10^−7^ ∼ 10^−9^ M). However, phosphorylated antigens can be pulsed on APCs, indicating that they are bound by BTN3A1 under assay and physiological conditions. Direct binding between BTN3A1 and Vγ9Vδ2 T cells was specific and weak in the absence of IPP. However, BTN3A1 and Vγ9Vδ2 T cells binding is facilitated by IPP engagement. This new role of BTN3A1 is far beyond co-stimulatory molecules in conventional αβ T cells as reported previously (35). Human Vγ9Vδ2 T cells are critical components of protective anti-microbial and anti-cancer immunity that involve cells that upregulate IPP. Therefore, it is of great value to understand how BTN3A1 modulates Vγ9Vδ2 cells in inflammatory diseases and cancer patients. Therapeutic effect of targeting BTN3A1 may depend on the function of Vγ9Vδ2 in specific diseases. Therapeutic targeting of BTN3A1 may potentially expand the bandwidth of immune regulation in clinic.

### Function of BTN/BTNL in APC

In addition to co-inhibitory function of BTNL1 T cell immunity, a recent study focused on the function in enterocytes (42). BTNL1 is highly expressed in small intestine, particularly on all enterocytes in close juxtaposition with T cells. When an enterocyte-derived cell line is transfected with *Btnl1*, CD8αβ^+^TCRαβ^+^ and CD8αα^+^γδ intraepithelial lymphocytes-induced cytokine production was inhibited in a cell–cell contact-independent manner. The cytokines include IL6, CXCL1, CCL4, and IL15. However, monocyte chemoattractant protein-1 (MCP1), IFNγ, and CCL2 remained unchanged by *Btnl1* overexpression. As epithelial cells are protective sites where infection occurs frequently, the capability of BTNL1 to dampen the response of enterocytes to activated intraepithelial lymphocytes may be essential for leveraging colitis and colon cancer. The close juxtaposition between BTNL1 and cell receptor suggested that the interaction between intraepithelial lymphocytes and enterocytes plays an essential role in sustaining the integrity of colon tissues. Nevertheless, it remains unknown whether BTNL1 controls the development of colon inflammation *in vivo* or not. More *in vivo* studies using the specific deletion or overexpression of BTNL1 in enterocytes may facilitate the understanding and therapeutic targeting of BTNL1 in inflammatory diseases and tolerance induction.

### Clinical indication of BTN/BTNL and immune tolerance

The first clinical association between BTN/BTNL proteins and immune function modulation was identified between BTNL2 mutation and sarcoidosis (43). SNP analysis of 947 independent and familial sarcoidosis cases identified a G–A transition constituting rs2076530, leading to a mutant protein lacking the C-terminal IgC domain and transmembrane helix. The mutant protein fails to localize on the cell membrane and loses co-inhibitory function, leading to over-activated T cells and thus overt inflammation. Several other studies reported the increased susceptibility to autoimmune sarcoidosis and myositis in patients bearing BTNL2 mutations (44). In addition, studies also showed correlation between BTNL2 polymorphism and ulcerative colitis (45–47), tuberculosis (48), RA, and systemic lupus erythematosus (49). However, more validation studies are required to establish the direct correlation between BTNL2 polymorphism and disease progression or severity. Going from bench to bedside, more efforts should be taken to understand the role of different BTN/BTNL family members in modulating the function of specific immune cells in various inflammatory diseases and organ transplantation.

Like some B7 family members, different BTN family members can also interact with unknown binding partners in different cell types, eliciting inhibitory or stimulatory response. Thorough search for the binding partners of these BTN/BTNL members will certainly facilitate the therapeutic development of agonist or antagonist Ab or BTN-BTNL/Ig fusion protein in the organ transplant maintenance. However, as transplant rejection is rather complicated with various immune compartments involved, more preclinical studies are warranted with any potential therapeutic candidate. We will discuss below the current progress of manipulating the well-understood immune modulatory molecules in clinical development and what lessons we can learn from the past.

## Transplant Tolerance in Clinical Practice

Transplant tolerance is still challenging in clinical practice, although immunosuppression drugs, such as corticosteroids, have been widely used in transplantation practice. Orlando et al. summarized that out of 461 liver transplantation patients, 163 were successful in maintaining the graft during immunosuppression drugs withdrawal (50). On the contrary, there is only one case report of operational tolerance in lung transplant (51) and one in heart transplant (52).

To meet the clinical needs for tolerance maintenance, different treatments have been developed by depleting effector B and T cells, and enhancing the suppressive capability of Tregs and tolerant DCs. Clinical trials are undergoing through depletion of T cells using a-CD52 (53) and B cell depletion by anti-CD20 (Rituximab) (NCT00568477). Recent studies also explored the efficacy of Treg adoptive transfer (NCT02088931) and bone marrow transplantation (54). To target effector cells and “regulatory” cells simultaneously, some immunomodulatory molecules seem practical based on the current data available in cancer studies. Below, we will discuss specifically the drug development of check-point regulators in clinic trial and what this means for future work.

Numerous studies are testing the clinical feasibility of targeting co-stimulatory or co-inhibitory molecules to treat both autoimmune diseases and prolong organ transplant survival. The earliest and most well-understood pathway is CD28/CTLA4–CD80/CD86 pathway. In this regard, CTLA4-Ig fusion protein (abatacept) was the first immune check-point regulator drug approved for the treatment of RA with good safety profile (5, 6). Abatacept binds to both CD80 and CD86, thus inhibiting both co-stimulatory CD28 and co-inhibitory CTLA4 signaling pathway. Early clinical trials of abatacept showed no significant benefit in symptoms of mild atopic asthma (55), multiple sclerosis (56), or ulcerative colitis (57). However, a second generation of CTLA-Ig fusion protein (belatacept) has a lower disassociation rate from CD80 and CD86 (58). In a Phase III trial of renal transplantation, patients treated with belatacept and calcinurin inhibitor showed superior function of renal transplants and multiple other clinical parameters (7, 59). However, belatacept-treated patients also experienced a higher incidence and severity of acute rejection episodes 1 year after transplantation than those patients treated with calcinurin inhibitor only.

Further studies focused on antagonistic CD28 Ab (sc28AT) to block the interaction between CD28 and CD80/CD86 without disturbing the CTLA4 pathway. Non-human primate studies suggested that the combination therapy of sc28AT and calcineurin inhibitor induced CTLA4-dependent decrease of T cell function and Treg promotion in the heart allografts in macaques (60). In addition to the CD28/CTLA4 pathway, the CD40/CD154 pathway has resurged as promising target in the clinic. Non-human primate studies showed that non-depleting CD40 blocking Ab showed efficacy in delaying rejection of bone marrow, islet (61), and renal transplantation. Thus, a Phase IIa clinical trial is underway to measure the efficacy of anti-CD40 (ASKP1240) in renal transplantation (NCT01780844). The clinical trials of targeting these pathways are summarized in Table 1.

**Table 1:** **Summary of the key clinical trials of targeting immune modulatory molecules and specific immune cells to gain immune tolerance**.

Test agent	Target	Clinical trial number	Trial phase	Disease area	Reference
Campath-1H (alemtuzumab)	Anti-CD52	NCT00365846	Phase II	Renal transplantation	(53)
Rituximab	Anti-CD20	NCT00568477	Phase II	Renal transplantation	N/A
Treg transfer	Treg	NCT02088931	Phase I	Renal transplantation	N/A
Abatacept	CTLA4-lg	NCT00048568, NCT00420199	Phase III	Rheumatoid arthritis	(5)
Abatacept	CTLA4-lg	NCT00784459	Phase II	Mild atopic asthma	(55)
Abatacept	CTLA4-lg	NCT01116427	Phase I/II	Multiple sclerosis	(56)
Abatacept	CTLA4-lg	NCT00410410	Phase III	Ulcerative colitis	(57)
Belatacept	CTLA4-lg	NCT00114777	Phase II	Renal transplantation	(7, 59)
		NCT00346151	Phase III	
ASKP1240	Anti-CD40	NCT01780844	Phase II a	Renal transplantation	N/A

As we learned from past experiences, different molecules may contribute to various aspects of allograft rejection, through resting, activated, or memory T cell function regulation. Therefore, more effort should be taken to understand the intercrossing signaling pathways mediated by different molecules on APCs or T cells. This will facilitate the identification of the appropriate pathway to target with least side effects. The novel B7 family members and BTN/BTNL members may play different roles than other “old” molecules at different development stages of various cell types. For instance, the high expression of B and T lymphocyte attenuator (BTLA) in γδ T cells (62) may suggest that this molecule is important for modulating the pro-inflammatory activities of γδ T cells. More studies are warranted to determine whether co-inhibitory molecules on γδ T cells play a significant role in inflammatory diseases and allograft rejection.

The manipulation of both co-stimulatory and co-inhibitory molecules in the clinic may bring promising outcomes in control of tolerance maintenance. The incoming data from on-going and future clinical trials will shed light on how different co-inhibitory molecules can be engaged to tune down the host response against the donor transplant. More characterization is required to understand the specific impact of these drugs in facilitating Treg generation and inhibiting effector function. The selection of therapeutic Ab isotypes and Ig fusion proteins takes thorough exploration to determine the best therapeutic efficacy with least toxicity. On the other hand, better understanding of the co-stimulatory molecules will facilitate their function analysis in tolerance maintenance during organ transplantation. The antagonistic Ab against the costimulatory molecules may block the effector T and/or B cell function by directly interfering with the ligand–receptor binding on T cells. Alternatively, these molecules can also function via interrogating the suppressive capability of myeloid cells. More studies are needed to address the different functions of these specific molecules in effector and regulatory cells.

## Concluding Remarks

The discovery of various new Ig superfamily member proteins has facilitated the clinical treatment of cancer, and tolerance maintenance in inflammatory diseases and organ transplant. The increasing effort on preclinical studies and drug development toward these molecules will eventually determine the best single agent or combinatorial approach with most desirable efficacy and least toxicity. In our view, in-depth understanding of these molecules in the following aspects is critical for clinical benefits. First, in preclinical studies, it is of great importance to identify the unknown binding partners and the down-stream signaling pathways mediated by engagement of both co-inhibitory and co-stimulatory molecules. The identification of binding partners for known molecules, such as VISTA and B7-H4, is essential for understanding the mechanism of antagonistic or agonistic Ab/Ig fusion protein in altering the function of various immune cells. Shared receptor(s)/ligand(s) among different Ig superfamily molecules is not unusual, so the identification of binding partners for new molecules will also help to determine the affected pathways. The intracellular signaling pathway mediated by Ig superfamily proteins engagement is critical for the assay development during Ab or Ig fusion protein screening in drug development. The recently established function of these molecules in inflammatory cells, such as γδ T cells, offers a different angle to target these molecules. Second, clinical studies need to focus on the following aspects to better harness the immune system in tolerance maintenance across different diseases. It is important to establish the correlation between inflammation and BTN/BTNLs, and other novel B7 family members in patients, such as RA, transplant rejection, and so on. From on-going clinical trials, extensive analysis of known immune check-point regulators may shed new light on the optimal target molecules/immune cells. The identification of biomarkers is critical for both the assessment of efficacy and the understanding of mechanism of therapeutic agents. Finally, in inflammatory diseases and organ transplantation treatment, combinatorial approaches targeting Ig superfamily members with current standard of care regimes may potentially offer enhanced therapeutic advantage. However, the complexity of the disease severity and the transplant recipients need to be evaluated carefully before determining the treatment regime due to the potential danger of long-term immunosuppression. In conclusion, more studies will allow for better understanding both the mechanism of these novel molecules in preclinical studies and clinical trials for best development of therapeutic agents in various diseases.

## Conflict of Interest Statement

Yanxia Guo and Adele Y. Wang are employed by Merck Inc. and Co.
