# Erratum: Novel Immune Check-Point Regulators in Tolerance Maintenance

**DOI:** 10.3389/fimmu.2016.00038

**Published:** 2016-02-08

**Authors:** Frontiers Production Office

**Affiliations:** ^1^Frontiers Production Office, Frontiers, Switzerland

**Keywords:** B7, butyrophilin, immune tolerance

**Reason for Erratum:**

Due to an error, a citation of Pawar et al. (1), referenced in the last paragraph of section B7-H4, was missing from the Reference List. The full reference should be:

Pawar RD, Goilav B, Xia Y, Herlitz L, Doerner J, Chalmers S, et al. B7x/B7-H4 modulates the adaptive immune response and ameliorates renal injury in antibody-mediated nephritis. *Clin Exp Immunol* (2015) **179**:329–43. doi: 10.1111/cei.12452

The publisher apologizes for this mistake.

This error does not change the scientific conclusions of the article in any way.

## Conflict of Interest Statement

The authors declare that the research was conducted in the absence of any commercial or financial relationships that could be construed as a potential conflict of interest.
